# Cylindrical Implant Versus Tapered Implant: A Comparative Study

**DOI:** 10.7759/cureus.29675

**Published:** 2022-09-28

**Authors:** Navari Nandini, Ramesh Kunusoth, Aditya Mohan Alwala, Rathod Prakash, Shalini Sampreethi, Saideep Katkuri

**Affiliations:** 1 Department of Oral and Maxillofacial Surgery, Manthena Narayana Raju (MNR) Dental College and Hospital, Sangareddy, IND

**Keywords:** osstell isq instrument, edentulous mandible, secondary stability, primary stability, resonance frequency analysis, conical implants, parallel walled implants, implant stability quotient (isq)

## Abstract

Aims and objectives: The aim of this study is to compare the efficacy of tapered implants with cylindrical implants by evaluating the implant stability using the Osstell implant stability quotient (ISQ) instrument (W&H Dental India PVT Ltd., Bangalore, India) postoperative pain using the visual analog scale (VAS) score, and the peri-implant health using implant mobility scale.

Materials and methods: This study included 30 patients who were partially edentulous with single or bilateral missing teeth and received 30 tapered implants on the one side and 30 cylindrical implants on the other side. Implant stability, postoperative pain, and peri-implant health were evaluated.

Results: In the evaluation of 30 tapered implants and 30 cylindrical implants, the implant stability quotient (ISQ) value for the tapered implants was higher when compared to that of cylindrical implants, and the group that received tapered implants had the least pain and good peri-implant health than the group that received cylindrical implants.

Conclusion: On the basis of our clinical findings, it can be concluded that tapered implants provide greater primary stability than cylindrical implants. With the popularity of loading protocols in implant dentistry, the implant surgeon can increase predictability and success by selecting a tapered implant.

## Introduction

Missing teeth may deteriorate esthetics, mastication, and speech. Dental implants are the most widely used fixed restorations for partially or completely edentulous patients. Because of their esthetic and functional advantages, dental implants have more than 90% survival rates [1]. The success of implants depends on implant stability, peri-implant health, and also postoperative pain. For the success and survival of dental implants, attaining implant stability is mandatory [2].

Implant stability plays a crucial role in successful osseointegration. Osseointegration is the direct structural and functional connection between living bone and the surface of a load-bearing dental implant [3]. Implants attain stability at two different stages: primary and secondary. Primary stability is the mechanical engagement of the implant with the cortical bone. Secondary stability is the result of remodeling and regeneration of the bone and tissue around the implant. Since implant stability is dependent on the condition of the surrounding tissues, it should be possible to evaluate implant stability over time and predict the long-term prognosis using implant stability as a basis [4]. Implant stability (primary and secondary) is influenced by bone quality and quantity, surgical instrumentation, and implant macro- and micro-design features [5]. Over the years, implant macro- and micro-structures have been modified to improve both primary and secondary stability by maximizing the contact of implant surface area with the surrounding bone and better engagement of the marginal cortical and lateral bone [6].

Implant geometry and design are one of the main features that ensure implant success. Cylindrical and tapered implants are the two major designs that differ in the healing sequence following implant placement [7]. They consist of different components of compressive load at the implant-bone interface, which depends on the degree of taper [8].

Tapered implants create tight contact between the osteotomy wall and the implant surface. This provides excellent primary stability but causes localized bone necrosis at the implant surface before bone apposition ensures its biomechanical fixation. Cylindrical implants were less stable at the time of implant placement but rapidly gained stability due to early woven bone formation following the blood-clotted gap between the implant surface and osteotomy wall [9].

In tapered geometry, forces get diverted from the dense cortical bone to the resilient trabecular bone, which leads to higher forces in the apex. Whereas in cylindrical geometry, force load is distributed throughout the implant and, due to the parallel walls of the cylindrical implant, the coronal part of the osteotomy will be damaged by the preceding implant threads [10]. In the literature, tapered implants show a greater possibility of attaining initial fixation than cylindrical implants [2].

Peri-implant health is assessed based on the visible plaque index, presence of calculus, degree of peri-implant inflammation, gingival bleeding index, and probing depth index [11].

Along with the stability and peri-implant health, the pain after implant surgery is also of concern for the surgeon and the patient for a satisfactory treatment outcome [12]. Pain after implant placement is usually mild to moderate, but sometimes exceeds the normal range. The level of pain depends on surgical difficulty, the surgeon’s expertise, and the patient’s pain threshold and anxiety. When compared with cylindrical implants, the order of drilling and bone removal in tapered implant systems causes less trauma [13].

This study is to compare the efficacy of cylindrical implants versus tapered implants by evaluating implant stability, peri-implant health, and postoperative pain and to provide evidence for appropriate implant selection.

## Materials and methods

The study included thirty patients who were partially edentulous and reported to the department of oral and maxillofacial surgery for the need to replacement of their teeth using implants. Informed consent was obtained from all the patients.

Inclusion criteria

(1) Patients above the age of 18 years to 60 years, both males and females. (2) Patients with the bilateral partially edentulous mandible. (3) Patients who are periodontally and systemically healthy without any underlying diseases affecting bone healing and fit for implant therapy. (4) Patients with the presence of adequate quality and quantity of native bone or grafted bone. (5) Patients with the presence of sufficient zones of keratinized tissue. (6) Patients are willing to participate in this study by giving written consent.

Exclusion criteria

(1) Patients below the age of 16 years. (2) Patients who are pregnant and lactating mothers. (3) Patients with uncontrolled medical or mental conditions, periodontal diseases, or the need for systemic antibiotics for endocarditis prophylaxis, and known drug allergy. (4) Patients with active infection and inflammation in the areas intended for implant placement. (5) Patient has taking bisphosphonates for the last 12 months. (6) Patients who are chronic smokers. (7) Patients with any neuromuscular disorders such as peripheral neuropathy, myopathy, and multiple sclerosis. (8) Patients who do not consent to the procedure.

Patients who fulfilled the inclusion criteria were selected and quadrants with single missing teeth were randomly divided into two groups. Group 1: Quadrants with missing teeth receive 30 tapered implants. Group 2: Quadrants with missing teeth receive 30 cylindrical implants.

Radiographic examination

Standardized intra-oral periapical (IOPA) radiographs and orthopantomograms were taken to assess bone architecture and surrounding structures. The obtained image allowed for precise evaluation of the available bone height above the mandibular canal for the selection of implant length. Ridge mapping was done using a bone caliper to determine the alveolar ridge width for the selection of implant diameter.

Surgical procedure

Immediately before surgery, the patient was advised to rinse with 0.12% chlorhexidine mouthwash. Under local anesthesia infiltration sub-periosteally (2% lignocaine with 1:80,000 adrenaline), a crestal incision was given followed by complete mucoperiosteal flap reflection for delayed loading implants. The implant bed preparation was started with a standard pilot drill under copious internal and external irrigation of chilled saline. The angulation of the osteotomy drill was checked using a paralleling tool. Sequential drilling was done using standard drills with the help of physio dispensers. The longest and widest pre-selected implants were threaded into the prepared site up to crestal level using a ratchet with an insertion tool. All implants showed good primary stability after the placement of the implant. Here, the implant mount was removed with the help of a hex tool, followed by the placement of the cover screw.

All implants used are Genesis (JJ Implants, Munipara, Kerala). The abutment is separate, and it is the receiving area for the prosthetic component of restoration. An implant is placed at the level of the crest of bone. The implant's length ranges from 10 to 13 mm and its diameter from 4.2 to 5 mm. After implant placement, the soft tissue edges were sutured to protect the implant sites for delayed-loading implants.

Antibiotics (tablet amoxyclav 625 mg BD for five days) and anti-inflammatory medication (tablet aceclofenac + paracetamol + serratiopeptidase BD for three days) were prescribed for all patients. Sutures were removed after seven days, and patients were reviewed on a regular basis. Standardized intra-oral periapical radiographs were taken to assess the peri-implant radiolucency.

The second stage surgical procedure was performed three months after the first procedure. The implant was exposed using a biopsy punch to remove the cover screw and to place a gingival healing cap. After two weeks of the healing abutment, the implant was restored with a metal-ceramic single crown prosthesis. Patients were reviewed for assessment of implant stability quotient (ISQ), implant mobility, and postoperative pain caused by the implants during the period of healing.

Parameters used for evaluation

*Implant Stability* 

**Figure 1:**
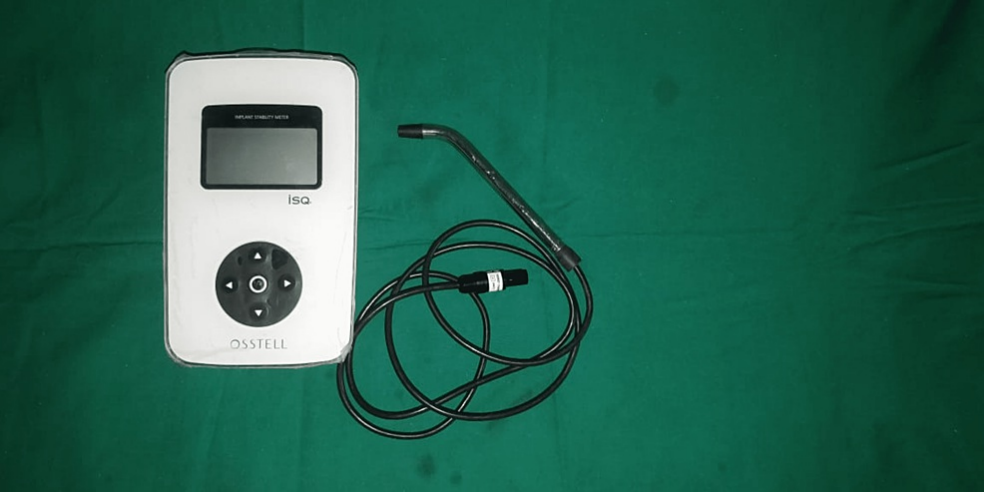
Osstell ISQ instrument. ISQ: implant stability quotient.

The implant stability was assessed using the Osstell ISQ instrument (W&H Dental India PVT Ltd., Bangalore, India) (Figure 1). It works on the principle of resonance frequency analysis, and the implant stability is measured in the form of ISQ, whose values reveal a frequency range of 6 to 9 kHz, with the ISQ value in the range of 0 to 100. There is a probe that is attached to the device at the tip, and this probe is touched to the smart peg to receive the resonance frequencies. A smart peg assembly is selected from the different smart peg sizes available with the Osstell implant stability quotient apparatus. 

**Figure 2:**
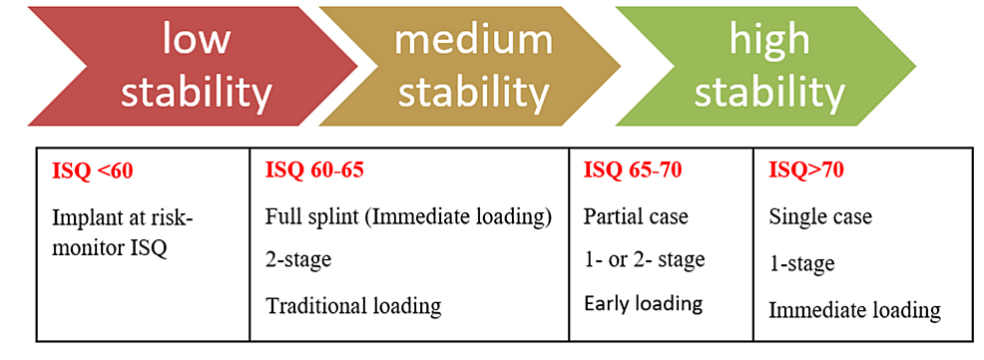
ISQ scale. ISQ: implant stability quotient.

The ISQ values for all the implants were recorded on the day of implant placement and at the first, third, and sixth months post-implant placement. ISQ values are related to implant stability. A higher ISQ value indicates rigid fixation of the implant (Figure 2).

Implant Mobility

It was measured with two rigid instruments. A force of approximately 500 g was applied in the labiolingual direction. The amplitude of implant mobility was scored 0-4, according to Table 1. 

**Table 1:** Clinical implant mobility scale by Carl E. Mish.

Scale	Description
0	Absence of clinical mobility in any direction
1	Slight detectable horizontal movement
2	Moderate visible horizontal mobility up to 0.5 mm
3	Severe horizontal movement greater than 0.5 mm
4	Visible moderate to severe horizontal and any visible vertical movement

Postoperative Pain

**Figure 3:**
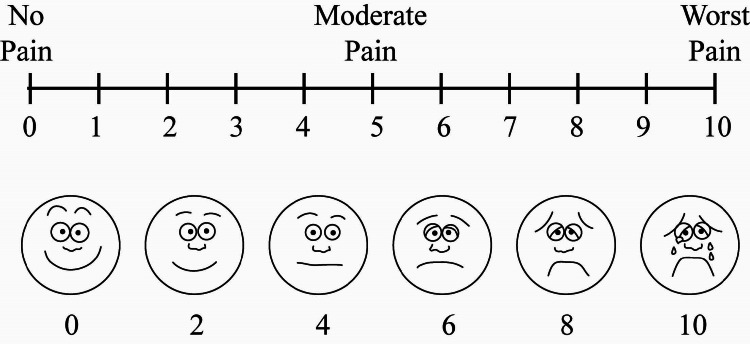
Visual analog scale.

Postoperative pain was assessed using the visual analog scale (VAS) (Figure 3).

The patient makes a mark on the line to represent their current state of perception. The oral and maxillofacial surgeon interpreted the score as no pain, moderate pain, and worst pain.

## Results

In this study, a total of 60 implants were placed in a total of 30 patients. Thirty tapered implants were placed in group 1, and 30 cylindrical implants were placed in group 2, in the lower posterior teeth region.

The data were analyzed with the Statistical Package for Social Sciences (IBM SPSS Version 20.0). The descriptive data show the mean and standard deviation (SD) were used for comparison between the groups.

Immediately following the placement of the implant, the mean implant stability was significantly higher in group 1 (66.9±1.02) in comparison to group 2 (62.7±1.31; p=0.000). After the first month of follow-up, the mean implant stability was significantly higher in group 2 (72.6±1.32, p=0.000). However, at the third and sixth-month follow-up, the mean stability was significantly higher in group 1 (77.2±0.90, 81.2±0.90, respectively) in comparison to group 2 (75.2±0.96; 79.3±0.95, respectively) (Table 2).

**Table 2:** Mean comparison of implant stability between groups at all levels of follow-up. Kruskal-Wallis test; *P<0.05 considered statistically significant.

Follow-up		N	Mean	SD	P-value
Immediately	Group 1	30	66.9000	1.02889	0.000*
	Group 2	30	62.7333	1.31131	
One month	Group 1	30	70.6000	1.22051	0.000*
	Group 2	30	72.6000	1.32873	
Three months	Group 1	30	77.2667	0.90719	0.000*
	Group 2	30	75.2000	0.96132	
Six months	Group 1	30	81.2667	0.90719	0.000*
	Group 2	30	79.3000	0.95231	

**Figure 4:**
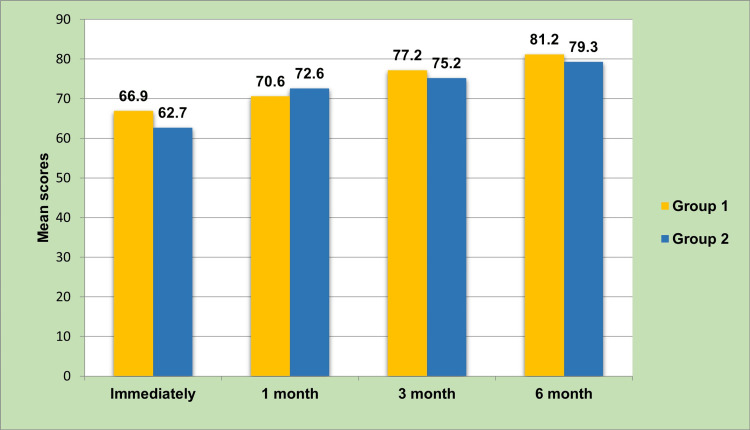
Mean comparison of implant stability between groups at all levels of follow-up.

Implant stability between group 1 and group 2 immediately, after one month, three months, and six months of follow-up is compared in Figure 4.

It was observed that at all levels of follow-up, the mean VAS score was significantly higher among group 2 (2.66±0.6, 4.66±1.63, 4.1±0.75, and 2.6±0.72, respectively) in comparison to group 1 (1.5±0.5, 2.36±0.49, 1.63±0.49, and 0.8±0.4, respectively) (Table 3).

**Table 3:** Mean comparison of VAS between groups at all levels of follow-up. VAS: visual analog scale. Kruskal-Wallis test; *P<0.05 considered statistically significant.

Follow-up		N	Mean	SD	P-value
Three hours	Group 1	30	1.5000	0.50855	0.000*
	Group 2	30	2.6667	0.60648	
Six hours	Group 1	30	2.3667	0.49013	0.000*
	Group 2	30	4.6667	0.71116	
Twelve hours	Group 1	30	1.6333	0.49013	0.000*
	Group 2	30	4.1000	0.75886	
Twenty-four hours	Group 1	30	0.8000	0.40684	0.000*
	Group 2	30	2.6000	0.72397	

**Figure 5:**
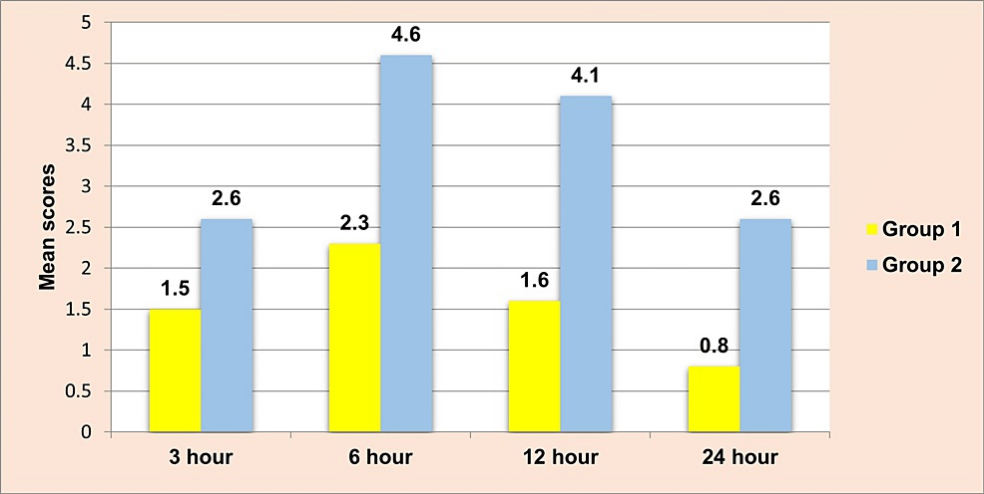
Mean comparison of VAS between groups at all levels of follow-up. VAS: visual analog scale.

The VAS between group 1 and group 2 after three hours, six hours, twelve hours, and twenty-four hours of follow-up is compared in Figure 5.

It was observed that the group 2 subjects who received cylindrical implants showed mobility in more numbers than in comparison to group 1 at all levels of follow-up (Table 4).

**Table 4:** Comparison of mobility between groups at all levels of follow-up. Chi-square test; *P<0.05 considered statistically significant.

Follow-up		Present		Absent		P-value
		N	%	N	%	
One month	Group 1	1	3.3	29	96.7	0.833
	Group 2	5	16.7	25	83.3	
Three months	Group 1	2	6.7	28	93.3	0.193
	Group 2	3	10	27	90	
Six months	Group 1	0	0	30	100	-
	Group 2	4	13.3	26	86.7	

**Figure 6:**
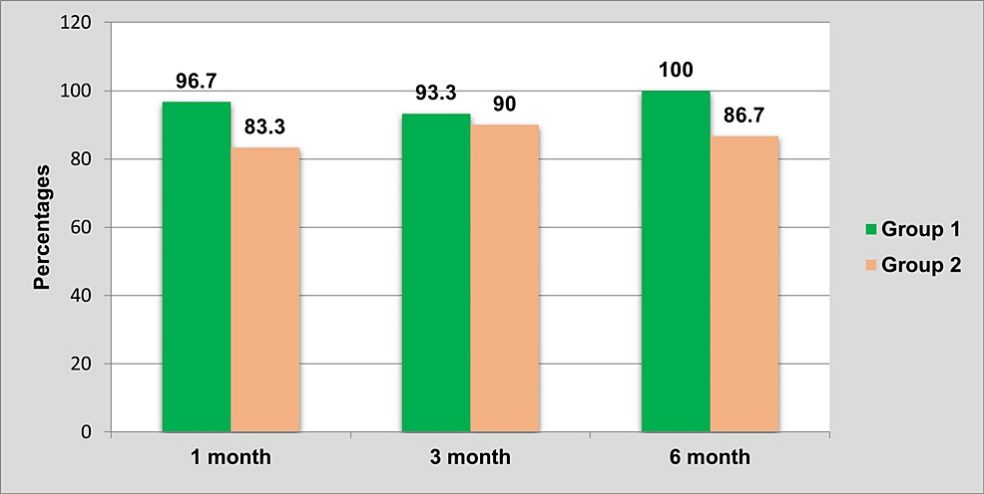
Comparison of the absence of mobility between groups at all levels of follow-up.

The absence of implant mobility between group 1 and group 2 after one month, three months, and six months of follow-up is compared in Figure 6.

**Figure 7:**
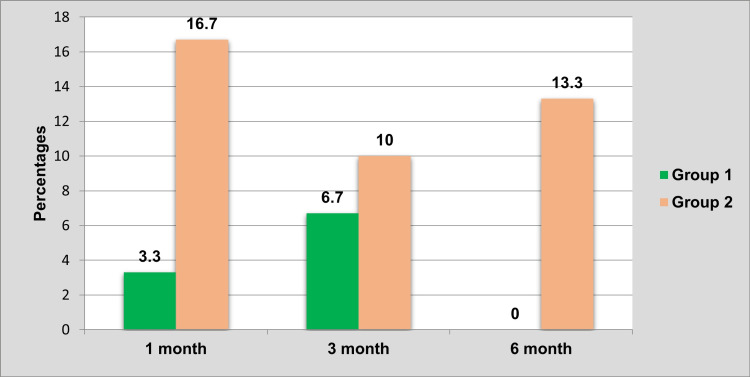
Comparison of the presence of mobility between groups at all levels of follow-up.

The presence of mobility between group 1 and group 2 after one month, three months, and six months of follow-up is compared in Figure 7.

## Discussion

Contemporary dentistry is creative as well as therapeutic. Modern dentistry aims at rehabilitating the patient's form, function, and esthetics of their lost dentition with a near resemblance to natural teeth. Rehabilitation of lost teeth can be attained by various methods, which include fixed or removable prostheses. In fixed prosthesis itself, there are two types, fixed partial denture and dental implants. Nowadays, people have become more aware of the merits of dental implants as they are more comfortable and do not need to be removed. Such an advanced treatment that rehabilitates the lost tooth with proper form, function, esthetics, comfort, near resemblance to natural teeth, and conserving the adjacent tooth are known as dental implants.

Successful integration of dental implants is predominantly dependent on the stability of the dental implant following placement. Dental implant stability is a measure of the anchorage or lack of movement of the implant in the alveolar bone. Implant stability can be divided into two different stages: primary and secondary [4]. Selecting an implant that provides adequate primary stability in the bone bed is essential to achieving clinical success. Primary stability depends on bone quality, surgical technique, and implant design [14]. Similar primary implant stabilities for tapered and cylindrical dental implants were also reported in vitro by Sakoh and colleagues [15].

The prosthodontic glossary defines primary stability as “contributing factors of mechanical stabilization of a dental implant during the healing phase” [16]. Basically, it is the initial stability or fixation of a dental implant immediately after placement within the bone [17]. Secondary stability is crucial for successful osseointegration. Secondary stability is achieved as additional bone is remodeled at the implant interface. If sufficient primary stability is not initially achieved, secondary stability will be compromised [4].

Dental implant design has been shown to be a crucial parameter for attaining primary stability. Implant design directly influences implant handling of biomechanical forces. Tapered and cylindrical-walled implants distribute biomechanical forces differently. Tapered implants divert forces toward the apex, making this type of implant more desirable for immediate placement. Parallel-walled implants distribute forces throughout the entire implant because the parallel walls of the osteotomy are damaged by the preceding implant threads.

In a prospective clinical trial, Lozano-Carrascal et al. compared osseospeed (OSP) implants to tapered MIS® implants (MIS®, Medical Implants System, Israel) in human mandibles. They found that tapered implants achieved higher primary stability measured through ISQ and insertion torque [18].

Other similar studies have utilized RFA analysis and artificial bone blocks or animal models (bone from cow ribs) and concluded that tapered implants show significantly higher ISQ values than cylindrical-shaped implants [19,20]. An additional advantage of tapered implants is that they require a lesser quantity of bone at their apex due to a decreased apical diameter. Therefore, they are less likely to perforate the buccal plate when osseous undercuts are present.

In this study, all the patients were evaluated for clinical parameters, such as implant stability, implant mobility, and postoperative pain. Each parameter was evaluated with the help of a scoring system on every visit of the patient.

In our study, the implant stability among the sample that received tapered implants revealed that the mean implant stability significantly increased at each follow-up (p=0.000).

The above results are similar to the multicentre randomized-controlled clinical trial done by Lang et al. 2007 in which he demonstrated increased ISQ values for tapered implants compared to cylindrical implants. In a prospective clinical study, Lozano-Carrascal et al. compared cylindrical implants to tapered MIS® implants placed in human mandibles. They reported the tapered implants achieved higher primary stability measured through ISQ and insertion torque [13-18]. In our study, we found higher mean ISQ values in tapered implants when compared to cylindrical implants.

George et al. (2015) conducted a study to compare implant survival and success as an outcome of two types of implant morphology, either the tapered or cylinder forms, in the delayed immediate placement of dental implants [10]. This study concluded that tapered implants showed better primary stability than straight-walled cylindrical implants and had a higher success rate.

Implant mobility is an important clinical parameter that warrants continual assessment during the maintenance period. In our study, implant mobility was assessed using a clinical implant mobility scale at the time of implant placement. When both the groups are compared, it was observed that group 2 (cylindrical implants) showed mobility in more numbers than in comparison to group 1 (tapered implants) at all levels of follow-up.

Waechter et al. conducted a study to compare the clinical outcomes of tapered and cylindrical implants and to study their effects on bone site characteristics and peri-implant health during healing [11]. This study concluded that tapered and cylindrical implants have similar biological behavior during the healing process.

Implant surgery usually causes mild to moderate and rarely severe pain, depending on the duration and difficulty of surgery, bone quality, amount of trauma to the bone and soft tissue, and a patient’s tolerance and reaction. The pain is assessed using a visual analog scale (VAS), which is a psychometric response scale that can be used in questionnaires. This study shows that the group that received tapered implants had the least pain when compared to the group that received cylindrical implants.

The above results are in accordance with the study done by Samieirad et al., who conducted a study compared the level of postoperative pain between tapered and cylindrical implants inserted in the posterior region of the maxilla, in which he concluded that implant shape had an impact on postoperative pain and showed that tapered implants produced less stress and required smaller amounts of bone removal, resulting in less postoperative pain. In addition, the sequence of drilling and bone removal for tapered implants has been found to cause less trauma compared with the cylindrical system. Moreover, the amount of bone removed during drilling and bone compression during implant insertion might influence pain levels after implant surgeries [13]. The results of the present study follow the same pattern as the above studies.

Implant shape always has an impact on postoperative pain. In our study, two different types of implants were selected and compared to know the impact on postoperative pain. The resonance frequency analysis (RFA) technique provides clinically relevant information about the state of the implant-bone interface at any stage after implant placement. The implant stability quotient (ISQ) value reflects the micromobility of an implant when loaded, which in turn is determined by the biomechanical properties of the surrounding bone tissue and the quality of the bone-implant interface. It is likely that ISQ measurements can be used as one additional parameter for the diagnosis of implant stability and decision-making during implant treatment and follow-up.

For a better consistency of the presented evidence, it is suggested that larger cohort and multicentre clinical trials should be performed to know the efficacy of tapered versus cylindrical implants.

## Conclusions

On the basis of our clinical findings, it can be concluded that tapered implants provide greater primary stability than cylindrical implants. This is important because primary stability is a key prerequisite for successful osseointegration. Tapered implants have minimal postoperative complications and require less space in the apical region. They need minimal apical bone fenestration. Tapered implants can be easily placed in extraction sockets. With the popularity of loading protocols in implant dentistry, the implant surgeon can increase predictability and success by selecting a tapered implant.
